# Long-Lasting Inhibitory Effects of Fetal Liver Mesenchymal Stem Cells on T-Lymphocyte Proliferation

**DOI:** 10.1371/journal.pone.0019988

**Published:** 2011-05-19

**Authors:** Massimo Giuliani, Maud Fleury, Amelia Vernochet, Farah Ketroussi, Denis Clay, Bruno Azzarone, Jean Jacques Lataillade, Antoine Durrbach

**Affiliations:** 1 U1014 INSERM, Hôpital Paul Brousse, Villejuif, France; 2 U602 INSERM, Hôpital Paul Brousse, Villejuif, France; 3 Service de Nephrologie, Université Paris XI, Le Kremlin-Bicêtre, France; 4 IFR89, Hôpital Paul Brousse, Villejuif, France; 5 Laboratoire de Thérapie Cellulaire du CTSA (Centre de Transfusion Sanguine des Armées), Hôpital Percy, Clamart, France; University of Palermo, Italy

## Abstract

Human bone marrow mesenchymal stem cells (BM-MSC) are multipotent progenitor cells that have transient immunomodulatory properties on Natural Killer (NK) cells, Dendritic Cells (DC), and T cells. This study compared the use of MSC isolated from bone marrow and fetal liver (FL-MSC) to determine which displayed the most efficient immunosuppressive effects on T cell activation. Although both types of MSC exhibit similar phenotype profile, FL-MSC displays a much more extended *in vitro* life-span and immunomodulatory properties. When co-cultured with CD3/CD28-stimulated T cells, both BM-MSC and FL-MSC affected T cell proliferation by inhibiting their entry into the cell cycle, by inducing the down-regulation of phospho-retinoblastoma (pRb), cyclins A and D1, as well as up-regulating p27^kip1^expression. The T cell inhibition by MSC was not due to the soluble HLA-G5 isoform, but to the surface expression of HLA-G1, as shown by the need of cell-cell contact and by the use of neutralizing anti-HLA-G antibodies. To note, in a HLA-G-mediated fashion, MSC facilitated the expansion of a CD4^low^/CD8^low^ T subset that had decreased secretion of IFN-γ, and an induced secretion of the immunomodulatory cytokine IL-10. Because of their longer lasting in vitro immunosuppressive properties, mainly mediated by HLA-G, and their more efficient induction of IL-10 production and T cell apoptosis, fetal liver MSC could be considered a new tool for MSC therapy to prevent allograft rejection.

## Introduction

Mesenchymal stem cells (MSC) are multipotent stem cells able to form bone, cartilage and other mesenchymal tissues [1]. MSC can be isolated from several sources, including adult bone marrow and fetal tissues [2], [3], [4], [5], [ and 6]. Fetal and adult MSC share several common characteristics, including a fusiform fibroblast-like morphology and phenotype. The most accepted profile for MSC is a lack of expression of hematopoietic (CD34 and CD45) and endothelial (CD31) markers, and co-expression of CD105 (SH2), CD90 (Thy-1), CD73 (SH3), CD44 (HCAM), CD166 (ALCAM), and CD29 [1] as well as the recently described CD146 (MSCA-1) [7]. Bone marrow MSC (BM-MSC) and fetal MSC (FL-MSC) express human leukocyte antigen (HLA) class I molecules, but not HLA class II antigens, CD80 or CD86 co-stimulatory molecules. BM-MSC modulate hematopoiesis and can exert immune regulatory functions, both *in vivo* and *in vitro*, on a wide range of immunocompetent cells, including T cells [8], [9], [10], [11], [12], [13], [14], [15], NK cells [16], [17], [18], B cells [19], and dendritic cells (DC) [20], [21]. The immunomodulatory properties of BM-MSC require cell-cell contact as well as soluble factors, including interleukin (IL)-6 [22], hepatocyte growth factor (HGF) [10], nitric oxide (NO) [23], prostaglandin (PGE)-2 [17], indolamine 2,3 dioxygenase (IDO) [17], [24] and HLA-G [25], [26]. HLA-G is a non-classical MHC class Ib molecule with a low polymorphism, a restricted tissue distribution, and tolerogenic functions that contribute to fetal-graft tolerance via the maternal immune system and to human allograft acceptance [27]. Four membrane-bound (HLA-G1 to -G4) and three soluble (HLA-G5 to -G7) HLA-G isoforms have been described that inhibit immune cellular functions. HLA-G modulates immune responses by interacting with specific receptors ILT-2, ILT-4 and KIR2DL4 [28]. The mechanism of action of HLA-G is controversial: we have demonstrated that it inhibits cell-cycle progression of allogeneic T cells [29], whereas others have demonstrated that the HLA-G molecule induces T-cell apoptosis through the Fas pathway [30]. In addition, the immunomodulatory properties of HLA-G have been mainly attributed to the soluble isoform HLA-G5 [26]. *In vivo*, BM-MSC prevent graft-versus-host disease [9], [11], but they only delay for few days allograft rejection in animals given heart-transplants [31].

Consequently several groups have isolated MSC from other sources with the hope that these cells could display much more efficient immunoregulatory functions [3], [32]. In this study we have compared immunoregulatory properties of MSC derived from adult and foetal tissues. In addition, we have investigated the involvement of HLA-G in this process. Herein, we show that BM-MSC and FL-MSC express the membrane-bound HLA-G1 isoform that is directly involved in the inhibition of T cell activation. Moreover, both types of MSC blocked the entry into the cell cycle, and favored the generation of a CD4^low^/CD8^low^ T subset with an impaired IFN-γ secretion. Remarkably, FL-MSC displays much longer-lasting immunomodulatory properties compared to BM-MSC, and are more efficient at inducing T cell apoptosis and secretion of the immunosuppressive cytokine IL-10.

## Results

### Adult and fetal MSC share potential and immunosuppressive functions

**Figure 1:**
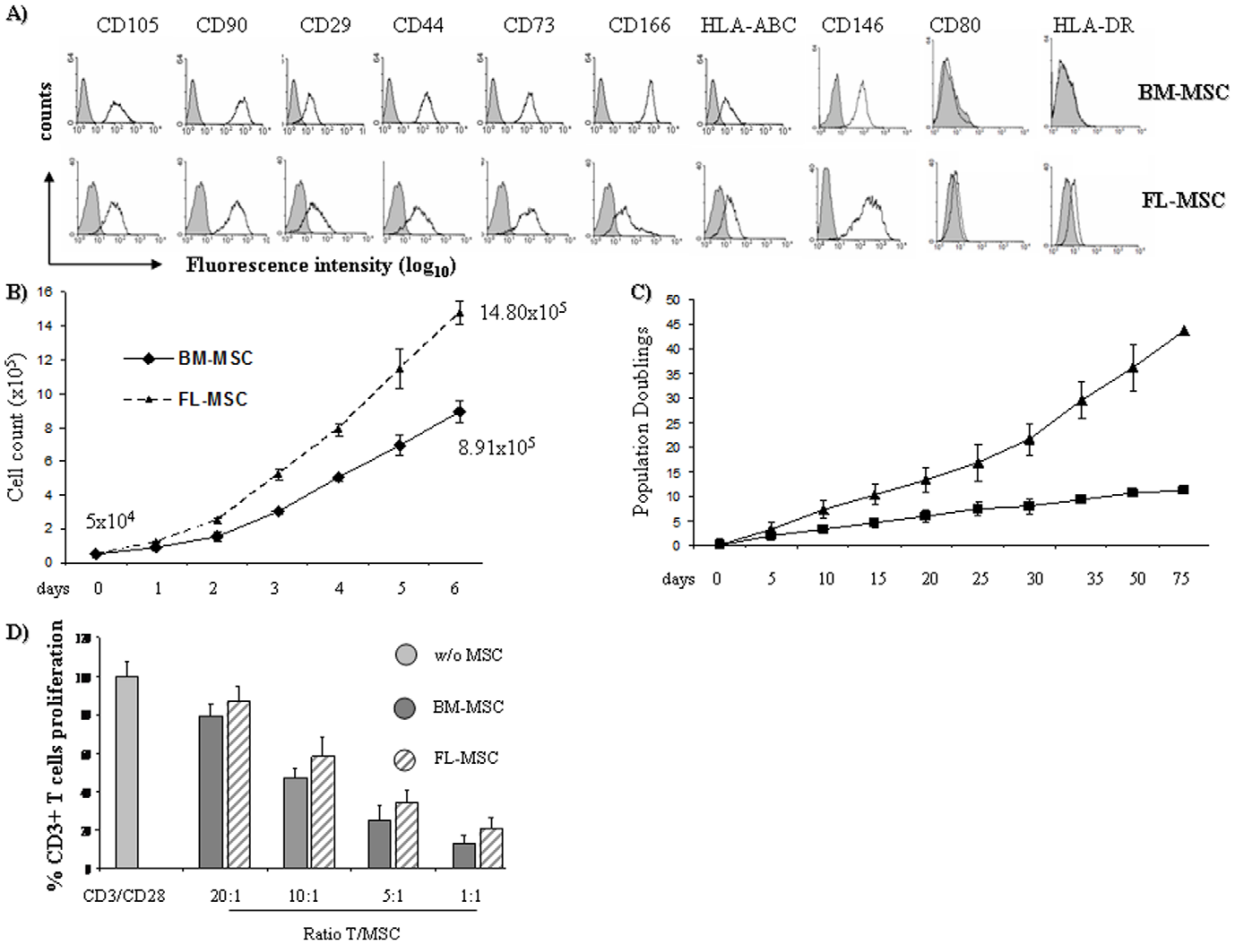
Characterization proliferation potential and immunosuppressive functions of adult and fetal MSC. (**a**) Bone marrow MSC (n = 6), cultured between passage 2 and 4, and human fetal-liver MSC, cultured between passage 12 and 18 (n = 5), were characterized by cell surface-marker expression using flow cytometry. The gray curves indicate the corresponding negative mouse IgG1 or IgG2a antibodies. Data corresponds to a typical staining of BM-MSC and FL-MSC. (**b**) Time course of cell growth in culture of BM-MSC and FL-MSC. 5×10^4^ of both MSCs were seeded in 6 wells plate (4×10^3^/cm^2^). (**c**) Cell count of MSC after 6 days. Averages of three independent experiments are shown. (**d**) Activated lymphocytes (stimulated with anti CD3 and anti CD28) were cultured in the presence of adult MSC (passage 2–4, n = 3) or fetal MSC (passage 12–15, n = 3) at different T∶MSC ratios (CFSE assay, 5 µM for 5 min at 37°C). Averages of six independent experiments are shown.

After 14–18 days of culture in specific medium, both adult (BM-MSC) and fetal MSC (FL-MSC) were all positive for CD90, CD105, CD44, CD29, CD166, CD146 and CD73 specific MSC markers (**Figure 1A**). They were negative for HLA class II and CD80 co-stimulatory molecules and had a multipotent ability to differentiate into adipocytes, chondrocytes, or osteocytes *(data not shown)*. To compare proliferation potential of fetal and adult MSC, we evaluated their cumulative population doubling over 40–50 days, by performing cellular counts at confluence at each cell sub-culture. Our results indicate that during 50 days BM-MSC had less than 10 doublings, while FL-MSC underwent about 30 doublings, showing that FL-MSC proliferate much faster than adult MSC (**Figure 1B-C**). In order to compare regulatory properties between adult bone marrow and fetal MSC, T cells were activated with CD3/CD28 and co-cultured at different effector/target (E/T) ratios (from 1∶20 to 1∶1) with BM-MSC or FL-MSC. CFSE assay was used to evaluate lymphocytes proliferation. In the absence of MSC, CD3/CD28-stimulated lymphocytes proliferated strongly. In contrast, FL-MSC or BM-MSC prevented their proliferation. This inhibition was dependent on the E/T ratio: the strongest effect was observed with a 1∶1 ratio (80% inhibition), but was still significant with a 1∶10 ratio (50% inhibition) (**Figure 1D**).

### HLA-G is expressed in adult and fetal MSC

**Figure 2:**
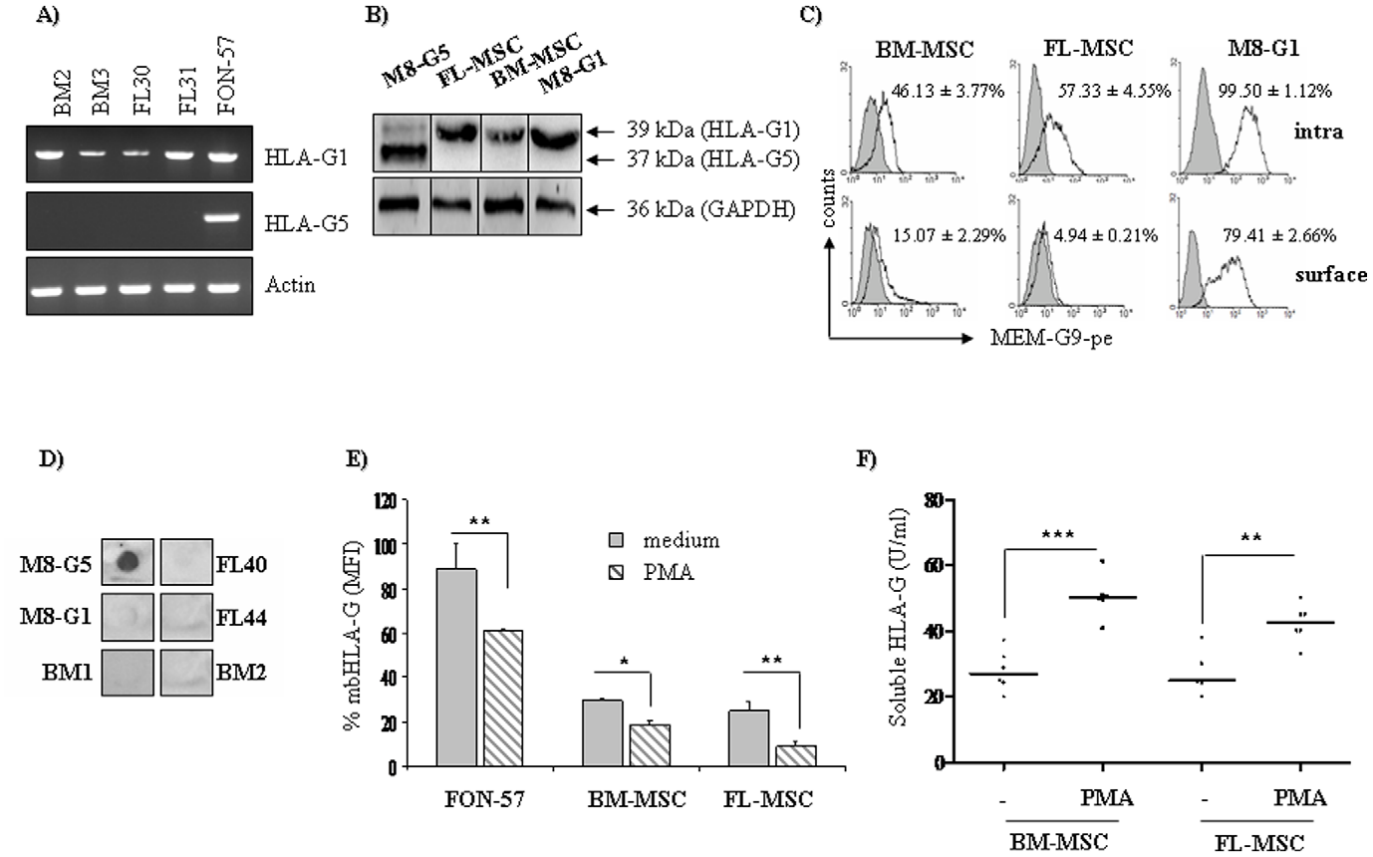
HLA-G expression in MSC. HLA-G was detected in BM-MSC and FL-MSC by using RT-PCR, western blot and flow cytometry. (**a**) The RT-PCR products from BM-MSC and FL-MSC, as a result of using specific primers for HLA-G1 and HLA-G5, were analyzed on an agarose gel. A FON-57 cell line and β-actin were used as positive controls for HLA-G1 and HLA-G5, and as a loading control, respectively. In other experiments, M8-G1 and M8-G5 transfected melanoma cell lines were used as positive control. (**b**) Detection of HLA-G isoforms in BM-MSC (passage 2–4, n = 3), FL-MSC (passage 12–15, n = 3), M8-G5 or M8-G1 (positive control) by western blot with 4H84 mAb. The 39 kDa bands correspond to HLA-G1 and the 37 kDa to the soluble form (the soluble HLA-G5 or the shed HLA-G1). (**c**) The same MSC were also stained at the cell surface or intracellularly with MEM-G/9-PE (filled histograms), a specific anti-HLA-G antibody. (**d**) Dot blot analysis showed that MSC do not secretes soluble HLA-G (clone 5A6G7), compared to melanoma M8-G5 cell line used as positive control. (**e**) To induce the shedding of the membrane bound HLA-G1, cells were treated or not with PMA (10 ng/ml) for 12 h and their HLA-G expression was evaluated by flow cytometry. PMA induce the release of HLA-G from M8-G1 cells as well as BM-MSC or FL-MSC, also confirmed by ELISA assay (**f**). Histogram indicated the percentage of decrease compared to the non treated cells (*P<0.01; **P<0.005). Averages of three independent experiments are shown.

The regulatory properties displayed by both adult and fetal MSC against T cells led us to investigate the mechanisms involved in this ability. Recent results have suggested that human MSC produce the immunoregulatory molecule HLA-G, which might participate to the regulation of T cell proliferation [33]. RT-PCR (**Figure 2A**) and western blot analysis (**Figure 2B**) showed that adult and fetal MSC express transcripts and protein for HLA-G1, but not for HLA-G5 (antibody 4H84, specific for both HLA-G1 and HLA-G5 isoforms). In addition, flow cytometry analysis (**Figure 2C**) showed that HLA-G can be expressed at both the cell surface and intracellularly by both types of MSC. Dot blot assay using the 5A6G7 antibody specific for HLA-G5, also shows that HLA-G5 isoform was not detected in supernatant of MSC (compared to M8-G5 melanoma cell line) (**Figure 2D**). Since soluble HLA-G has been reported to be released by MSC [33], these results suggest that membrane-bound HLA-G could be shedded and secreted from MSC. To investigate this hypothesis, we treated MSC with phorbol-myristate acetate (PMA), a powerful inducer of metalloproteases. As shown in **Figure 2E**, PMA-treated MSC displayed decreased HLA-G1 cell-surface expression, suggesting that HLA-G1 cell-surface expression is sensible to proteolytic shedding process dependent on metalloproteinase activity. PMA-induced down-regulation of membrane-bound HLA-G1 expression was accompanied by a significant increase of HLA-G in the supernatant after PMA treatment, as shown by an ELISA assay (**Figure 2F**).

### HLA-G plays a critical role in T cell proliferation mediated by MSC

**Figure 3:**
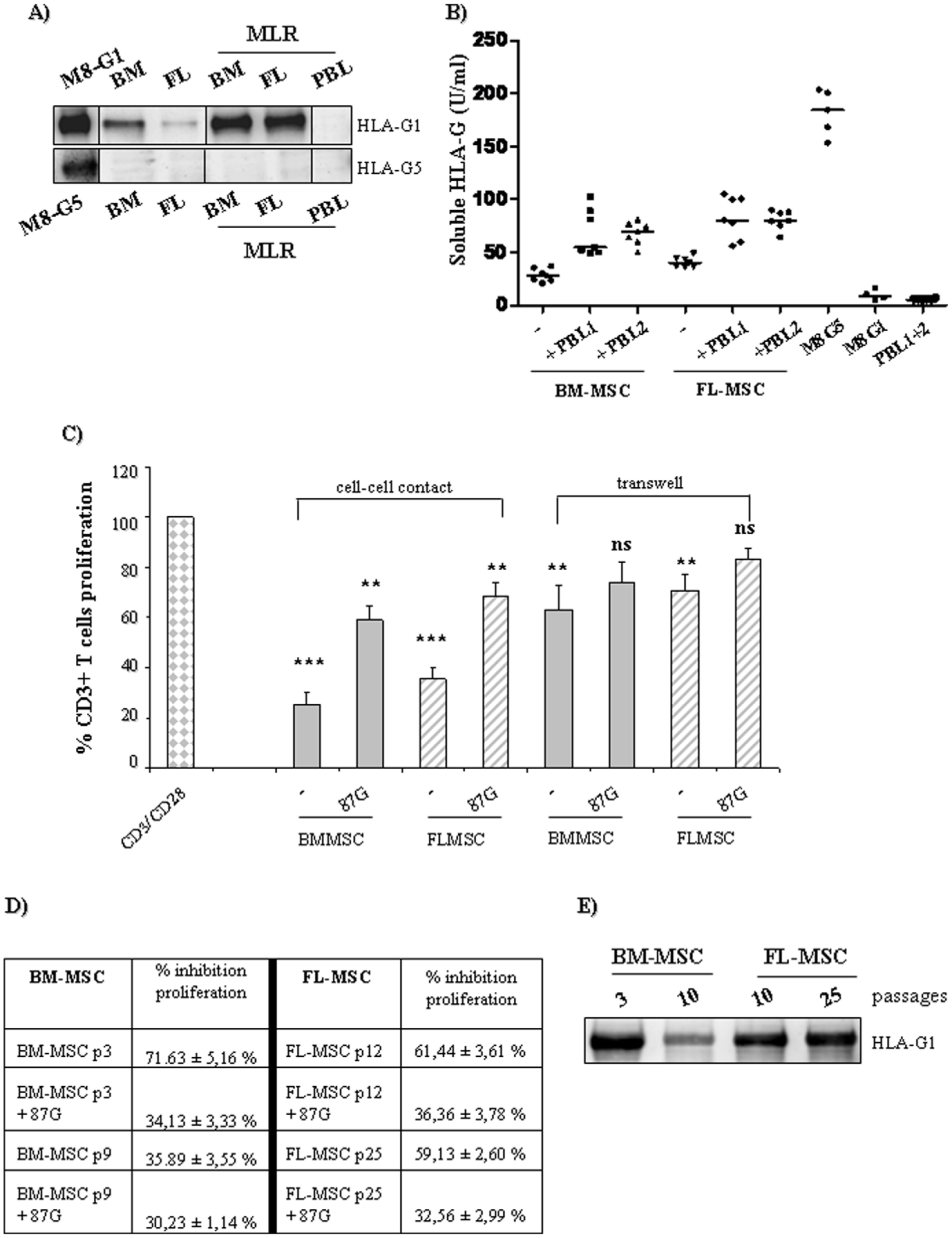
HLA-G plays a critical role in T cell-proliferation mediated by MSC. (**a**) Western blot analysis (4H84 and 5A6G7 mAbs) for HLA-G1 (39 kDa) and HLA-G5 (37 kDa) isoform after MLR (4 days). Following MLR, lymphocytes and MSC (passage 2–4 and 12–15 for adult and fetal cells, respectively) were loaded separately. (**b**) Soluble HLA-G released by MSC after MLR (4 days) was evaluated by ELISA assay. Two PBL and four adult or fetal MSC were used to evaluate HLA-G secretion. M8-G1 and M8-G5 melanoma cells were used as positive control for HLA-G1 and HLA-G5 isoform protein (contained and secreted, respectively. (**c**) CFSE-labeled T cells were cultured with MSC (ratio T∶MSC 5∶1) directly (cell-cell contact) or in transwell culture system. Neutralizing anti-HLA-G antibody (87G mAb) was added on days 0 and 2 (20 µg/ml), as previously reported [26]. (**d**) BM-MSC lacks immunomodulatory properties after more than 8 passages of culture, whereas FL-MSC have a more prolonged inhibitory effect on T cells proliferation which remains HLA-G dependant. (**e**) Western blot analysis of HLA-G1 (4H84 mAb) protein expression in BM-MSC (passage 3 and 10) compared to FL-MSC (passage 10 and 25). Averages of three independent experiments are shown. Error bars represent the SD. *P-*values were calculated using Student's t-test. *P<0.05, ** *P*<0.01; ***P<0.001, ns = not statistically significant.

We investigated whether HLA-G expressed by MSC could be modulated during MLR. Our results show that HLA-G1, but not HLA-G5, was strongly up-regulated in both MSCs during MLR (**Figure 3A**). PBL harvested from MLR did not express HLA-G1/5 protein. In addition, we show that soluble HLA-G, which was weakly secreted by both MSCs (<40 U/ml), was increased following MLR (**Figure 3B**). The inhibitory properties of MSC were mainly cell-contact-dependent, as transwell cultures induced a weaker inhibition of T cell proliferation compared to cell-contact cultures (**Figure 3C**). Moreover, the effect was HLA-G-dependent, since the addition of neutralizing antibody anti-HLA-G1/-G5 (87G mAb) restored lymphocyte proliferation in cell-contact cultures with both BM- and FL-MSC (from 71% to 34% and 61% to 36%, respectively). The effect of HLA-G was evaluated by using increasing dose of the blocking HLA-G mAb (87G) both on BM-MSC and FL-MSC during MLR. The higher restoration of T cell proliferation was observed by adding 20 µg/ml of the HLA-G blocking antibody (87G) (**Figure S1**). The addition of 87G mAb to transwell cultures did not significantly restore T cell proliferation, highlighting that other soluble factor than HLA-G could be involved in MSC suppressive functions. Moreover, the addition of the isotype control IgG2A has no impact on the inhibitory properties of MSC (**Figure S1**). In addition, we observed that BM-MSC lost their immunoregulatory properties after 6–8 passages (40–50 days in culture), whereas FL-MSC maintained their immunoregulatory functions for at least 25 passages (75–90 days in culture) Noteworthy, the decrease of MSC's immunosuppressive properties was associated with a down-regulation of HLA-G expression in aged BM-MSC (passages>9). In contrast, FL-MSC maintained HLA-G expression even after passage 30 (**Figure 3E**). By starting with 5×10^4^ cells, 8–10×10^6^ BM-MSC with preserved immunoregulatory functions can be produced (before passage 9–11). In the same time interval more than 30–35×10^6^ FL-MSC can be produced, and more than 55–70×10^6^ FL-MSC with preserved regulatory properties (before passage 30–35). These results show that fetal MSC during their lifespan much more immunoregulatory cells and display longer lasting regulatory abilities compared to adult MSC.

### Modulation of surface phenotype and cytokines secretion on T cells mediated by MSC

**Figure 4:**
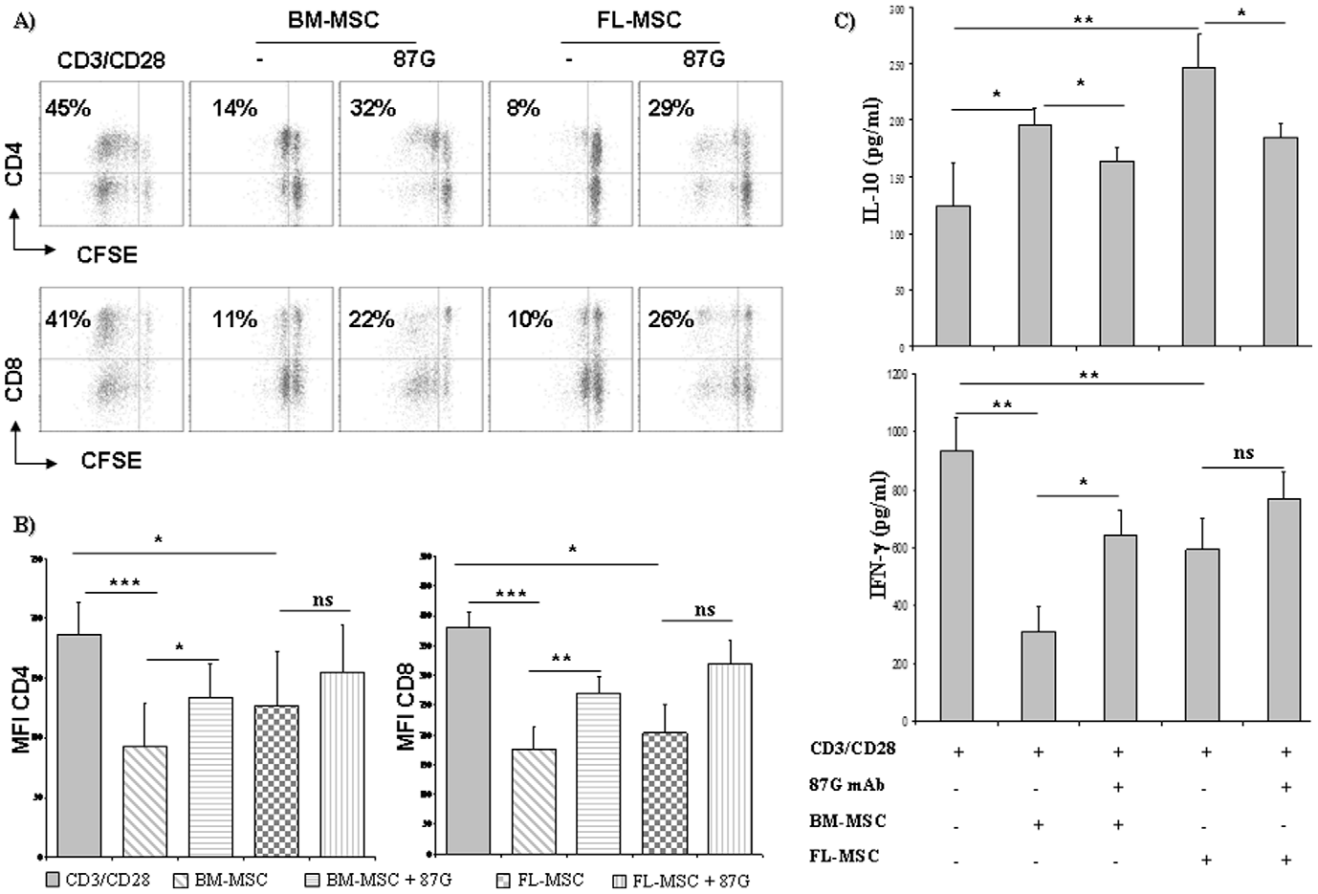
MSC facilitate a CD4^low^ and CD8^low^ subset expansion that secrete IL-10. (**a**) Surface CD4 and CD8 expression in CFSE-stained activated lymphocytes was evaluated by flow cytometry in the absence or presence of 20% adult and fetal MSC for 4 days. 87G anti-HLA-G antibody was added to evaluate the involvement of HLA-G. One representative experiment is shown. (**b**) CD4 and CD8 expression of T cells incubated with MSC (expressed as MFI, Mean Fluorescence Intensity) are presented. Error bars represent SD. *P-*values were calculated using Student's t-test. *P<0.05, **P<0.01; ***P<0.001, ns = not statistically significant. (**c**) CD3/CD28-activated T cells were cultured in the presence of BM-MSC and FL-MSC (passage 2–4 and 12–15 for adult and fetal cells, respectively) for 4 days and IL-4, IL-5, IFN-γ and IL-10 production was evaluated by using ELISA assay. Averages of four independent experiments are shown. *P-*values were calculated using Student's t-test. *P<0.05, **P<0.01; ***P<0.001, ns = not statistically significant.

We also asked whether MSC could modulate the phenotypic and cytokine profile of activated lymphocytes. After MLR, we observed a significant decrease in the percentage of CD4+ and CD8+ T cells (**Figure 4A–B**) which was partially restored by the anti-HLA-G blocking antibody (87G). We did not observe significant modification of any other functional markers, such as CD69 or CD25 *(data not shown)*. In contrast, we have found that MSC strongly down-regulated IFN-γ production and increased IL-10 secretion in activated T cells (**Figure 4C**). The addition of 87G anti-HLA-G mAb inhibited IL-10 up-regulation mediated by adult and fetal MSC and efficiently restored IFN-γ secretion mediated by adult, but not fetal MSC. Other cytokines, such as IL-4 and IL-5, were not modulated by the presence of BM-MSC or FL-MSC *(data not shown)*.

### MSC inhibit entry into the T cell cycle

**Figure 5:**
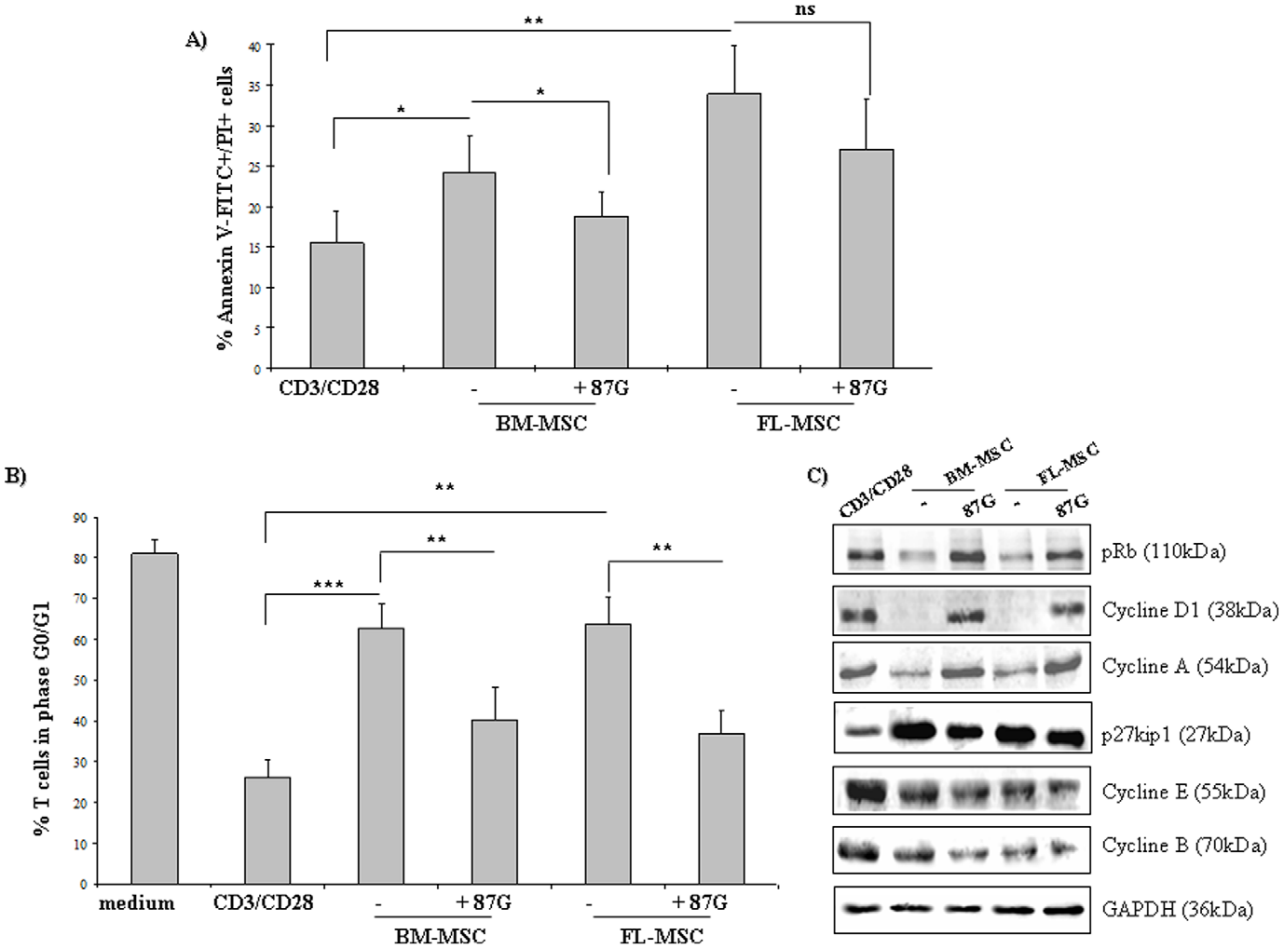
MSC regulates cell cycle entry of T cells, but do not induce cell apoptosis. Activated T cells were cultured with MSC (ratio T∶MSC of 5∶1) for 4 days. (**a**) Activated T cells were cultured with fetal and adult MSC to evaluate their apoptosis activities using the Annexin-V/PI assay. (**b**) Distribution of the G0–G1 phase in activated lymphocytes, as assessed by flow cytometry. (**c**) Expression of p27^Kip1^, pRb, and cyclins A, E, B and D1 were evaluated by western blot analysis. GAPDH was used as a loading control. Averages of four independent experiments are shown. Error bars represent SD. *P-*values were calculated using Student's t-test. *P<0.05, **P<0.01; ***P<0.001, ns = not statistically significant.

Because MSC inhibited T cell proliferation, we investigated the mechanism involved in this impairment. By using the Annexin V-FITC/PI (**Figure 5A**) and MTT assays *(data not shown)*, we found that BM-MSC did not significantly modify the level of apoptosis in activated T cells, whereas we observed a significant and HLA-G-independent increase in the number of apoptotic T cells after co-culture with FL-MSC. In addition, only 20% of activated lymphocytes were in G0/G1 phase, compared to the 80% of resting cells (**Figure 5B**). On the contrary, both adult and fetal MSC blocked activated cells in G0/G1 phase. The neutralizing anti-HLA-G antibody 87G strongly restored the T cell**s** proliferation. When we analyzed the activation status of the cyclin/cdk complex and the inhibitory kinase family during MLR, the level of phospho-retinoblastoma (pRb) was elevated in activated T cells, but was decreased when lymphocytes were co-cultured with BM-MSC or FL-MSC (**Figure 5C**). Similarly, the expression of cyclin D**1** and cyclin A were decreased in activated lymphocytes incubated with BM-MSC or FL-MSC when compared to fully activated lymphocytes. In contrast, MSC induced a strong up-regulation of p27^kip1^, an inhibitor of cyclin-dependent kinase involved in the regulation of the cell cycle. All these effects were inhibited when a neutralizing anti-HLA-G (87G) blocking antibody was added to the culture medium suggesting that HLA-G play a critical role in the immunomodulatory functions of BM-MSC and FL-MSC in regulating a balance between inhibitory molecules and proliferating signals. Furthermore, although decreased, the expression of cyclin E was not HLA-G-dependent, whereas cyclin B expression, which is predominantly expressed during the G2/M phase of the cell cycle, was not modified by MSC.

## Materials and Methods

### Ethics

Informed written approval was obtained from the INSERM institutional review board for these studies. Pregnant women gave written consent for the clinical procedure and for the use of blood or tissue for research purposes according to the Declaration of Helsinki.

### Cytokines, Abs, and reagents

The surface phenotype of MSC was assessed using specific FITC- or PE-conjugated monoclonal antibodies (mAb), such as CD29, CD44, CD80, HLA-DR, HLA-ABC (Immunotools), CD146 (Miltenyi), CD90, CD105, CD166 (R&D Systems), and CD73 (BD Pharmingen), whereas CD3 (BD Pharmingen), CD4, CD8, CD25, CD28, and CD69 (R&D Systems) were used to assess the T-cell surface phenotype. Anti-HLA-G (4H84, IgG1 anti-HLA-G1 recognizes an epitope present on the alpha-domain of the heavy chain) as well as anti-cyclin A/B1/E/D1 and retinoblastoma (Rb) protein antibodies were purchased from Santa Cruz Biotechnologies (Tebu, Le Parray en Yvelines, France). Anti-p27^kip1^ was purchased from BD Pharmingen. Neutralizing anti-HLA-G (87G, HLA-G1 and HLA-G5) and MEM-G/9 (native form of human HLA-G1) as well as the specific anti-HLA-G5 5A6G7 antibody were purchased from Exbio (Prague, Czech Republic). Recombinant human basic fibroblast growth factor (FGF) was obtained from Immunotools, whereas anti-CD28 was obtained from eBioscience. Mytomicin, Phorbol Myristate Acetate (PMA), and anti-CD3 (OKT-3) were obtained from Sigma.

### Generation of human adult and fetal mesenchymal stem cells

Human fetal livers (range 7–9 weeks of gestation, n = 7) were obtained from eight women after voluntary or therapeutic abortions. Informed written consent was obtained from the patient in accordance with the Declaration of Helsinki, and tissue collection and use were performed according to the guidelines and with the approval of the French Biomedicine Agency. Fetal liver cells were prepared as described previously [32]. Bone marrow samples were obtained from adult bone marrow aspirates (45±15 years, n = 6) to isolate MSC. Briefly, bone marrow and fetal mononuclear cells were isolated using a density Ficoll-Paque gradient separation and plated at 1.6×10^5^cells/cm^2^ in α-MEM (Life Technologies). This was supplemented with 2 mM L-glutamine, 1% penicillin/streptomycin solution (all from Gibco), and 10% heat-inactivated fetal calf serum (FCS; PAA Laboratories) (complete medium) at 37°C in air with 5% CO_2_. After three days, non-adherent cells were discarded and the cells were plated at 4×10^3^cells/cm^2^ in a α-MEM complete medium supplemented with bFGF (1 ng/ml). The medium was changed twice a week. When the cells were 70–80% confluent, the MSC were harvested by treating with trypsin-EDTA (Gibco) (passage 1) and were then cultured in a αMEM complete medium. Between passages 2 and 3, the MSC assumed a homogeneous morphology and were defined as cells positive for CD90, CD105, CD166, CD44, CD73, and CD29 MSC characteristic markers, and negative for CD45, CD14, and CD34. The bone marrow MSC at passage 2–4, and the fetal MSC at passage 12–18, which were used in this study, could be differentiated into chondrogenic, osteogenic, and adipogenic cells (R&D Systems). To compare fetal and adult MSC proliferation potential, their growth kinetics were evaluated by counting cell numbers after each passage and by estimating the cumulative population doubling over 30 days, as previously described [4].

### Cell isolation, mixed lymphocytes reaction (MLR) and suppression assays

T cells were isolated from healthy volunteers (n = 10) (Etablissement Français du Sang: Hôpital S. Louis, Paris, France) and cultured (1×10^6^ cells/ml) for 4 days with mitomycin-treated (25 µg/ml, 30 min, 37°C) MSC, and were used as responder cells to assess their proliferation. Briefly, freshly isolated T cells were resuspended in PBS/FCS 5% and labeled with 5 µM 5,6 carboxyfluorescein diacetate succinimidyl ester (CFSE, Molecular Probes, Eugene, OR) for 5 min at 37°C, as previously described [34]. T cells stimulated with anti-CD3 (OKT-3, 500 ng/ml) and anti-CD28 (1 µg/ml), in RPMI 1640 complete medium, were used as a control for T-cell proliferation. Activated lymphocytes were also co-cultured, with or without mitomycin-treated MSC, in 24-well flat-bottom plates (ratio T/MSC, 5∶1) for 4 days to evaluate marker expression by flow cytometry. The biological effects of HLA-G were assessed at days 0 and 2 by adding a specific neutralizing antibody (87G, 20 µg/ml, Exbio). T cell apoptosis was evaluated with Annexin V-FITC/PI assay (Beckman Coulters).

### Cell lines

The HLA class I-positive M8 and FON melanoma cell line were kindly provided by E. Carosella (Hôpital Saint-Louis, Centre HAYEM-CEA, Paris, France). M8 cells were transfected with a full-length HLA-G5 cDNA (M8-HLA-G5) or HLA-G1 cDNA (M8-HLA-G1) subcloned in vector pcDNA (Invitrogen Life Technologies) and used as positive control for HLA-G5 and HLA-G1 expression, respectively. Stable cell lines were selected with Hygromycin (50 µg/ml) (Invitrogen Life Technologies) in complete medium, as previously described [35], [36].

### Flow cytometry analysis

For surface-marker expression, cells were washed twice with PBS and, after Fc receptors were blocked, the cells were stained with saturating concentrations of the appropriate mAbs or isotype-matched Ab control for 30 min at 4°C, in the dark. Thereafter, cells were washed twice with PBS and kept at 4°C until analysis. For indirect fluorescence, FITC-conjugated anti-mouse was used as a secondary Ab (30 min at 4°C in the dark). For HLA-G intracellular staining, cells were permeabilised using a Cytoperm/Cytofix kit (BD Pharmingen). Cell cycle was determined in activated lymphocytes by washing cells with PBS and fixing them in 70% ethanol at −20°C until use. Fixed cells were washed with PBS and incubated with 0.5 µg/ml RNase A and 50 µg/ml propidium iodide for 30 min. Cells were analyzed using FACScan™ (BD Biosciences, Franklin, NJ, USA), with CellQuest Analysis (BD Biosciences) and FlowJo software (TreeStar).

### Western Blotting

After 4 days of MLR, T cells were analyzed for cell cycle protein expression. HLA-G in both MSCs was detected with an anti-HLA-G1/G5 antibody (clone 4H84) or with an anti-HLA-G5 (clone 5A6G7). Cells were lysed and the pellets (30 µg/lane) were resuspended in SDS-PAGE sample buffer, and analyzed using 7–12% SDS-PAGE. The resolved proteins were transferred onto nitrocellulose membranes (Bio-Rad), which were then saturated for 1 h and incubated with the specific monoclonal antibody in TBS-0.1% Tween-20. Membranes were washed three times in TBS-0.01% Tween-20, and were then incubated with HRP-conjugated goat anti-mouse IgG (Biotest Diagnostics, 1∶1500). An ECL detection system (ECL kit; Amersham Biosciences, Orsay, France) was used according to the manufacturer's instructions. Quantification of the protein blotted was determined using Fujifilm Intelligent Dark Box II and Image Gauge version 4.0 software.

### RT-PCR analysis for human HLA-G

Total RNA was isolated from MSC pellets using RNeasy Micro kit (Qiagen, France), according to the manufacturer's instructions, and quantified using a Nanodrop ND-100 (Nanodrop technologies, CA). One microgram of total RNA was incubated for 30 min at 37°C with 0.2 U RQ1 DNAse (Promega, Madison, WI) in reverse transcriptase (RT) buffer (Gibco, Life Technologies) in a final volume of 20 µl. M-MLV RT (Gibco) was added, and the mixture was further incubated for 1 h at 37°C. The volume of cDNA was then adjusted up to 200 µl. cDNA were separately amplified in a total volume of 50 µl containing 200 µM each dNTP (Invitrogen), 5 ng/µl of each primer, and 2 U of Taq DNA polymerase (Eppendorf, Germany). PCR was carried out with a Mastercycler *epgradient S* (Eppendorf) for 35 cycles (1 min at 94°C, 1 min at 57°C, 1 min at 72°C) with a final extension at 72°C for 10 min. 5 µL of sample was used with primers specific for β-actin and 10 µl with primers specific for HLA-G isoforms, as previously described [37].

### Cytokine detection by an enzyme-linked immunosorbent assay (ELISA)

Cultured cell supernatants were harvested after 4 days of co-culture and tested in triplicate for production of IFN-γ, IL-4 and IL-10 (eBioscience), HLA-G5 (clone 5A6G7) and total HLA-G (both from Exbio) and IL-5 (R&D Systems) by ELISA, according to the manufacturer's instructions. The ELISA plates were read at OD450 on a Microplate ELISA reader (Titertek multiskan plus, Puteaux, France).

### Data analyses

All experiments were performed in at least three independent assays, which yielded highly comparable results. Data were presented as mean +/− SD. The statistical significance (*P* values) of the results was calculated using the two-tailed Student's *t-*test. A *P*<0.05 was considered statistically significant.

## Discussion

BM-MSC have recently emerged as a therapeutic tool in regenerative therapy and transplantation. Their use is based on their plasticity and immunoregulatory properties. Because of their hypoimmunogenicity and their potential to create an immunosuppressive local microenvironment through the production of TGF-β, PGE-2, and HLA-G molecules [8], [38], BM-MSC are successfully employed to prevent graft-versus-host disease (GvHD) [11], [39], [40]. However, because of their transient immunoregulatory properties in transplantation models [26], we and others have attempted to identify new populations of MSC that can exert longer-lasting immunoregulatory properties [3]. Our data show that fetal liver MSC could be a good alternative to BM-MSC. Indeed, even though adult and fetal MSC exhibit a similar morphology and phenotype, FL-MSC are also characterized by: 1) faster growing kinetics than bone marrow MSC (30 vs. 8 doublings, respectively) with higher number of cells produced over the same period, 2) a prolonged synthesis of HLA-G and 3) a longer inhibition of T cell proliferation (>30 passages) compared to BM-MSC (<8 passages).

It has been previously reported that the *in vitro* life span of human fibroblasts is inversely related to donor's age and that the *in vitro* aging process is much more rapid for adult than fetal fibroblasts [41]–[42]. Herein, on the basis of the total number of population doublings (PD) performed and the total number of cells produced, we observed that BM-MSC undergo *in vitro* aging much more rapidly than FL-MSC. However, the loss of HLA-G expression and the decrease of regulatory functions were not associated with a significant decrease of proliferation potential of these cells. In addition, we did not observe any up-regulation of p16^INK4A^, a marker of senescence, in BM-MSC until passage 10, suggesting that our cells did not undergo massive senescence process [43]. In contrast, fetal MSC maintained long-lasting immunoregulatory properties. These results are complementary with those of Guillot et al., who demonstrated that FL-MSC grow faster and have longer telomeres than adult MSC [4], which is associated with a longer cellular longevity [44]. These properties render FL-MSC very attractive for therapeutic purposes, for which a rapid expansion as well as a great number of cells and prolonged immunoregulatory properties are required.

Our data show that both MSC populations inhibit T cell proliferation, mainly through cell-cell contacts involving the membrane isoform of the immunosuppressive molecule, HLA-G1, but not the soluble HLA-G5 isoform [26], [33], [ and 45]. RT-PCR analysis clearly indicates that both fetal and adult MSC expressed HLA-G1, but not the HLA-G5 transcript. Moreover, western blot analysis performed on total cell lysate revealed expression of the HLA-G1-specific 39 kDa band, but not the HLA-G5-specific 37 kDa band. However, we observed that HLA-G1 protein can be shedded and released by MSC incubated with PMA, which activates metalloproteases. This may explain the presence of the 37 kDa band (molecular weight of shed HLA-G1) in a previous publication [26]. To note, we found increased HLA-G1 protein expression in both MSCs after MLR, whereas HLA-G5 isoform was not found at basal levels, or after MLR. The increase of HLA-G1 was more important for FL-MSC than adult MSC. ELISA assays revealed that both MSC secrete very low levels of HLA-G (shed HLA-G), but its concentration was increased by their co-culture with T cells. However, the increased amount of secreted HLA-G was not enough to significantly inhibit lymphocyte proliferation in transwell experiments. The implication of HLA-G in controlling T cell response was demonstrated by the restoration of T cell proliferation in the presence of a neutralizing anti-HLA-G antibody in cell-cell contact conditions but not in transwell system cultures, which displays a residual HLA-G-independent inhibitory activity on T cell function. Both fetal and adult MSC strongly impaired T cell proliferation mainly by preventing the early phase of T cell cycle entry, by inducing a down-regulation of molecules that trigger the G0–G1 transition, such as the pRb protein or the cyclins A and D1, or by up-regulating molecules that inhibit this process, such as the p27^kip1^ protein. These events are reversed in the presence of the HLA-G blocking antibody (87G mAb), whereas the down-regulation of cyclin E expression was HLA-G-independent. Importantly, cyclin B expression, which is predominantly expressed during the G2/M phase of the cell cycle, was not modified by MSC. This highlights the fact that both adult and fetal MSC principally block the early G0/G1 transition phase of activated T cells.

Interestingly, it has been reported *in vivo* that a subset of double-transplanted patients showed improved graft acceptance which was associated with a high serum level of HLA-G and the expression of regulatory T cells, which synthesized IL-10 and have a CD3^+^/CD4^low^ and CD3^+^/CD8^low^ phenotype [33], [46]. In our experimental model, both MSCs consistently facilitated the HLA-G-dependent appearance of CD3^+^/CD4^low^ and CD3^+^/CD8^low^ with impaired IFN-γ secretion. However, the decrease of IFN-γ secretion was less pronounced in FL-MSC. This could correlate with the lower expression of HLA-G in FL-MSC at the basal level (before MLR), suggesting that the inhibitory effect on the IFN-γ secretion could be delayed until HLA-G expression is sufficiently produced by MSC or depend on the production of other unknown factors by FL-MSC compared to adult MSC. Both MSCs induced IL-10 secretion, but only FL-MSC raised biologically relevant concentration. Interestingly, it must be noted that FL-MSC but not BM-MSC induced significant levels of T cell apoptosis through a HLA-G-independent mechanism.

In summary, our results provide evidence that FL-MSC represent an attractive alternative to adult BM-MSC in the prevention of allograft rejection since they display several distinguish features that render these cells immunotolerants.

## Supporting Information

Figure S1 **Evaluation of optimal concentration and specificity of anti-HLA-G 87G.** CFSE-labeled T cells were cultured with MSC (ratio T∶MSC 5∶1) for 4 days. a) Involvement of HLA-G was evaluated using neutralizing anti-HLA-G (87G mAb) added on days 0 and 2 titrated at different concentrations. (b) The specificity of the 87G was verified with the corresponding antibody isotype control (10 µg/ml). Optimal concentration of 87G anti-HLA-G was obtained at 20 µg/ml for both MSCs. Averages of three independent experiments are shown. Error bars represent the SD. P-values were calculated using Student's t-test. ns = not statistically significant.
(TIF)

## References

[1] Pittenger MF, Mackay AM, Beck SC, Jaiswal RK, Douglas R (1999). Multilineage potential of adult human mesenchymal stem cells.. Science.

[2] Campagnoli C, Roberts IA, Kumar S, Bennett PR, Bellantuono I (2001). Identification of mesenchymal stem/progenitor cells in human first-trimester fetal blood, liver, and bone marrow.. Blood.

[3] Gotherstrom C, West A, Liden J, Uzunel M, Lahesmaa R (2005). Difference in gene expression between human fetal liver and adult bone marrow mesenchymal stem cells.. Haematologica.

[4] Guillot PV, Gotherstrom C, Chan J, Kurata H, Fisk NM (2007). Human first-trimester fetal MSC express pluripotency markers and grow faster and have longer telomeres than adult MSC.. Stem Cells.

[5] in 't Anker PS, Noort WA, Scherjon SA, Kleijburg-van der Keur C, Kruisselbrink AB (2003). Mesenchymal stem cells in human second-trimester bone marrow, liver, lung, and spleen exhibit a similar immunophenotype but a heterogeneous multilineage differentiation potential.. Haematologica.

[6] In 't Anker PS, Scherjon SA, Kleijburg-van der Keur C, de Groot-Swings GM, Claas FH (2004). Isolation of mesenchymal stem cells of fetal or maternal origin from human placenta.. Stem Cells.

[7] Sorrentino A, Ferracin M, Castelli G, Biffoni M, Tomaselli G (2008). Isolation and characterization of CD146+ multipotent mesenchymal stromal cells.. Exp Hematol.

[8] Aggarwal S, Pittenger MF (2005). Human mesenchymal stem cells modulate allogeneic immune cell responses.. Blood.

[9] Bartholomew A, Sturgeon C, Siatskas M, Ferrer K, McIntosh K (2002). Mesenchymal stem cells suppress lymphocyte proliferation in vitro and prolong skin graft survival in vivo.. Exp Hematol.

[10] Di Nicola M, Carlo-Stella C, Magni M, Milanesi M, Longoni PD (2002). Human bone marrow stromal cells suppress T-lymphocyte proliferation induced by cellular or nonspecific mitogenic stimuli.. Blood.

[11] Le Blanc K, Rasmusson I, Gotherstrom C, Seidel C, Sundberg B (2004). Mesenchymal stem cells inhibit the expression of CD25 (interleukin-2 receptor) and CD38 on phytohaemagglutinin-activated lymphocytes.. Scand J Immunol.

[12] Ramasamy R, Tong CK, Seow HF, Vidyadaran S, Dazzi F (2008). The immunosuppressive effects of human bone marrow-derived mesenchymal stem cells target T cell proliferation but not its effector function.. Cell Immunol.

[13] Rasmusson I, Ringden O, Sundberg B, Le Blanc K (2003). Mesenchymal stem cells inhibit the formation of cytotoxic T lymphocytes, but not activated cytotoxic T lymphocytes or natural killer cells.. Transplantation.

[14] Rasmusson I, Uhlin M, Le Blanc K, Levitsky V (2007). Mesenchymal stem cells fail to trigger effector functions of cytotoxic T lymphocytes.. J Leukoc Biol.

[15] Tse WT, Pendleton JD, Beyer WM, Egalka MC, Guinan EC (2003). Suppression of allogeneic T-cell proliferation by human marrow stromal cells: implications in transplantation.. Transplantation.

[16] Sotiropoulou PA, Perez SA, Gritzapis AD, Baxevanis CN, Papamichail M (2006). Interactions between human mesenchymal stem cells and natural killer cells.. Stem Cells.

[17] Spaggiari GM, Capobianco A, Abdelrazik H, Becchetti F, Mingari MC (2008). Mesenchymal stem cells inhibit natural killer-cell proliferation, cytotoxicity, and cytokine production: role of indoleamine 2,3-dioxygenase and prostaglandin E2.. Blood.

[18] Spaggiari GM, Capobianco A, Becchetti S, Mingari MC, Moretta L (2006). Mesenchymal stem cell-natural killer cell interactions: evidence that activated NK cells are capable of killing MSCs, whereas MSCs can inhibit IL-2-induced NK-cell proliferation.. Blood.

[19] Corcione A, Benvenuto F, Ferretti E, Giunti D, Cappiello V (2006). Human mesenchymal stem cells modulate B-cell functions.. Blood.

[20] Beyth S, Borovsky Z, Mevorach D, Liebergall M, Gazit Z (2005). Human mesenchymal stem cells alter antigen-presenting cell maturation and induce T-cell unresponsiveness.. Blood.

[21] Jiang XX, Zhang Y, Liu B, Zhang SX, Wu Y (2005). Human mesenchymal stem cells inhibit differentiation and function of monocyte-derived dendritic cells.. Blood.

[22] Djouad F, Charbonnier LM, Bouffi C, Louis-Plence P, Bony C (2007). Mesenchymal stem cells inhibit the differentiation of dendritic cells through an interleukin-6-dependent mechanism.. Stem Cells.

[23] Sato K, Ozaki K, Oh I, Meguro A, Hatanaka K (2007). Nitric oxide plays a critical role in suppression of T-cell proliferation by mesenchymal stem cells.. Blood.

[24] Meisel R, Zibert A, Laryea M, Gobel U, Daubener W (2004). Human bone marrow stromal cells inhibit allogeneic T-cell responses by indoleamine 2,3-dioxygenase-mediated tryptophan degradation.. Blood.

[25] Nasef A, Mathieu N, Chapel A, Frick J, Francois S (2007). Immunosuppressive effects of mesenchymal stem cells: involvement of HLA-G.. Transplantation.

[26] Selmani Z, Naji A, Zidi I, Favier B, Gaiffe E (2008). Human leukocyte antigen-G5 secretion by human mesenchymal stem cells is required to suppress T lymphocyte and natural killer function and to induce CD4+CD25highFOXP3+ regulatory T cells.. Stem Cells.

[27] Carosella ED, Moreau P, Le Maoult J, Le Discorde M, Dausset J (2003). HLA-G molecules: from maternal-fetal tolerance to tissue acceptance.. Adv Immunol.

[28] Carosella ED, HoWangYin KY, Favier B, LeMaoult J (2008). HLA-G-dependent suppressor cells: Diverse by nature, function, and significance.. Hum Immunol.

[29] Bahri R, Hirsch F, Josse A, Rouas-Freiss N, Bidere N (2006). Soluble HLA-G inhibits cell cycle progression in human alloreactive T lymphocytes.. J Immunol.

[30] Fournel S, Aguerre-Girr M, Huc X, Lenfant F, Alam A (2000). Cutting edge: soluble HLA-G1 triggers CD95/CD95 ligand-mediated apoptosis in activated CD8+ cells by interacting with CD8.. J Immunol.

[31] Zhou HP, Yi DH, Yu SQ, Sun GC, Cui Q (2006). Administration of donor-derived mesenchymal stem cells can prolong the survival of rat cardiac allograft.. Transplant Proc.

[32] Gotherstrom C, Ringden O, Westgren M, Tammik C, Le Blanc K (2003). Immunomodulatory effects of human foetal liver-derived mesenchymal stem cells.. Bone Marrow Transplant.

[33] Rizzo R, Campioni D, Stignani M, Melchiorri L, Bagnara GP (2008). A functional role for soluble HLA-G antigens in immune modulation mediated by mesenchymal stromal cells.. Cytotherapy.

[34] Quah BJ, Warren HS, Parish CR (2007). Monitoring lymphocyte proliferation in vitro and in vivo with the intracellular fluorescent dye carboxyfluorescein diacetate succinimidyl ester.. Nat Protoc.

[35] Le Rond S, Le Maoult J, Creput C, Menier C, Deschamps M (2004). Alloreactive CD4+ and CD8+ T cells express the immunotolerant HLA-G molecule in mixed lymphocyte reactions: in vivo implications in transplanted patients.. Eur J Immunol.

[36] Rouas-Freiss N, Bruel S, Menier C, Marcou C, Moreau P (2005). Switch of HLA-G alternative splicing in a melanoma cell line causes loss of HLA-G1 expression and sensitivity to NK lysis.. Int J Cancer.

[37] Yao YQ, Barlow DH, Sargent IL (2005). Differential expression of alternatively spliced transcripts of HLA-G in human preimplantation embryos and inner cell masses.. J Immunol.

[38] Uccelli A, Moretta L, Pistoia V (2008). Mesenchymal stem cells in health and disease.. Nat Rev Immunol.

[39] Koc ON, Gerson SL, Cooper BW, Dyhouse SM, Haynesworth SE (2000). Rapid hematopoietic recovery after coinfusion of autologous-blood stem cells and culture-expanded marrow mesenchymal stem cells in advanced breast cancer patients receiving high-dose chemotherapy.. J Clin Oncol.

[40] Ringden O, Uzunel M, Rasmusson I, Remberger M, Sundberg B (2006). Mesenchymal stem cells for treatment of therapy-resistant graft-versus-host disease.. Transplantation.

[41] Hayflick L (1985). The cell biology of aging.. Clin Geriatr Med.

[42] Roobrouck VD, Ulloa-Montoya F, Verfaillie CM (2008). Self-renewal and differentiation capacity of young and aged stem cells.. Exp Cell Res.

[43] Shibata KR, Aoyama T, Shima Y, Fukiage K, Otsuka S (2007). Expression of the p16INK4A gene is associated closely with senescence of human mesenchymal stem cells and is potentially silenced by DNA methylation during in vitro expansion.. Stem Cells.

[44] Mikhel'son VM, Gamalei IA (2011). [Telomere shortening is the main mechanism of natural and radiation aging].. Radiats Biol Radioecol.

[45] Nasef A, Chapel A, Mazurier C, Bouchet S, Lopez M (2007). Identification of IL-10 and TGF-beta transcripts involved in the inhibition of T-lymphocyte proliferation during cell contact with human mesenchymal stem cells.. Gene Expr.

[46] Naji A, Le Rond S, Durrbach A, Krawice-Radanne I, Creput C (2007). CD3+CD4low and CD3+CD8low are induced by HLA-G: novel human peripheral blood suppressor T-cell subsets involved in transplant acceptance.. Blood.

