# Supplementary figures and images for: Long-Lasting Inhibitory Effects of Fetal Liver Mesenchymal Stem Cells on T-Lymphocyte Proliferation
Source: PLoS One. 2011 May 19;6(5):e19988. doi: 10.1371/journal.pone.0019988 (PMC3098287; doi:10.1371/journal.pone.0019988)

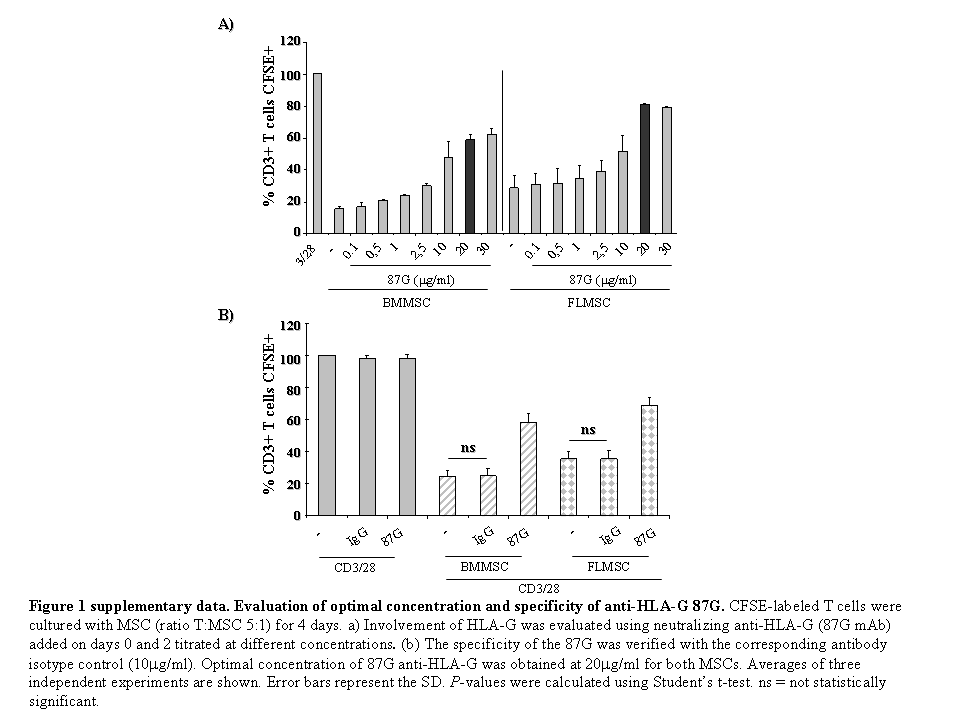

Supplement: Figure S1 — Evaluation of optimal concentration and specificity of anti-HLA-G 87G. CFSE-labeled T cells were cultured with MSC (ratio T∶MSC 5∶1) for 4 days. a) Involvement of HLA-G was evaluated using neutralizing anti-HLA-G (87G mAb) added on days 0 and 2 titrated at different concentrations. (b) The specificity of the 87G was verified with the corresponding antibody isotype control (10 µg/ml). Optimal concentration of 87G anti-HLA-G was obtained at 20 µg/ml for both MSCs. Averages of three independent experiments are shown. Error bars represent the SD. P-values were calculated using Student's t-test. ns = not statistically significant. (TIF) [file pone.0019988.s001.tif]
